# Putting research in place: an innovative approach to providing contextualized evidence synthesis for decision makers

**DOI:** 10.1186/s13643-017-0606-4

**Published:** 2017-11-02

**Authors:** Stephen Bornstein, Rochelle Baker, Pablo Navarro, Sarah Mackey, David Speed, Melissa Sullivan

**Affiliations:** Newfoundland and Labrador Centre for Applied Health Research, 95 Bonaventure Avenue, Suite 300, St. John’s, NL A1B 2X5 Canada

## Abstract

**Background:**

The Contextualized Health Research Synthesis Program (CHRSP), developed in 2007 by the Newfoundland and Labrador Centre for Applied Health Research, produces contextualized knowledge syntheses for health-system decision makers. The program provides timely, relevant, and easy-to-understand scientific evidence; optimizes evidence uptake; and, most importantly, attunes research questions and evidence to the specific context in which knowledge users must apply the findings.

**Methods:**

As an integrated knowledge translation (KT) method, CHRSP:

- Involves intensive partnerships with senior healthcare decision makers who propose priority research topics and participate on research teams;
- Considers local context both in framing the research question and in reporting the findings;
- Makes economical use of resources by utilizing a limited number of staff;
- Uses a combination of external and local experts; and
- Works quickly by synthesizing high-level systematic review evidence rather than primary studies.

Although it was developed in the Canadian province of Newfoundland and Labrador, the CHRSP methodology is adaptable to a variety of settings with distinctive features, such as those in rural, remote, and small-town locations.

**Results:**

CHRSP has published 25 syntheses on priority topics chosen by the provincial healthcare system, including:

- Clinical and cost-effectiveness: telehealth, rural renal dialysis, point-of-care testing;
- Community-based health services: helping seniors age in place, supporting seniors with dementia, residential treatment centers for at-risk youth;
- Healthcare organization/service delivery: reducing acute-care length of stay, promoting flu vaccination among health workers, safe patient handling, age-friendly acute care; and
- Health promotion: diabetes prevention, promoting healthy dietary habits.

These studies have been used by decision makers to inform local policy and practice decisions.

**Conclusions:**

By asking the health system to identify its own priorities and to participate directly in the research process, CHRSP fully integrates KT among researchers and knowledge users in healthcare in Newfoundland and Labrador. This high level of decision-maker buy-in has resulted in a corresponding level of uptake. CHRSP studies have directly informed a number of policy and practice directions, including the design of youth residential treatment centers, a provincial policy on single-use medical devices, and most recently, the opening of the province’s first Acute Care for the Elderly hospital unit.

## Background

The past two decades have seen increasing efforts by both health-system knowledge users and researchers to increase the use of research-based evidence in health-policy decisions. Researchers have been striving to improve the uptake of their work by decision makers in healthcare, while decision makers have become increasingly concerned about using research evidence more effectively in the development, implementation, and evaluation of health policies and programs [1]. Systematic reviews, health technology assessments (HTAs), and other research-based knowledge synthesis products have been introduced to provide support for evidence-informed decisions [2]. Various knowledge translation approaches have also been developed to build more effective working partnerships between researchers and decision makers. In Canada, such efforts to connect decision makers and researchers have included researcher-decision maker partnerships, policy-driven research funding programs, and the creation of organizations at the federal, provincial, regional, and hospital levels dedicated to supporting the use of evidence in health decision making [3]. Despite some progress, however, implementation of evidence-informed health programming across provinces/territories remains uneven and incomplete.

Research from Canada and other developed countries points to a variety of barriers to the uptake of evidence in health policy [4–7]. The challenge for decision makers to consider an ever-increasing number of health research publications is compounded by fiscal pressures that reduce the availability of specialized staff to carry out this work [8, 9]. While systematic reviews and health technology assessments have been heralded as a way to facilitate reception and uptake of health research evidence, these reports are often slow to produce, lengthy to read, too complicated to readily grasp, or not sufficiently attuned to local concerns, capacities, and needs to provide effective support for decision makers. A lack of easily identified and interpreted key messages in such reviews also complicates the decision-making process [10, 11].

The issue of timely access to evidence is also critical—research uptake must be coordinated within organizational deadlines for policy making [8–10, 12–15]. A further barrier to uptake is the perception among many decision makers that reviews and findings are not in step with their priorities nor attuned to the specific contexts in which decisions must be taken [16–20].

Applied health researchers aiming to support health system leaders and to see their work utilized within the health system must, therefore, seek to produce knowledge syntheses that combine several features rarely found together: their reports must be scientifically robust, accessible, timely, of direct concern to decision makers, and sensitive to the specific challenges and capacities facing those decision makers and the health systems they manage [5–7, 21].

Mini-HTAs, produced by individual hospitals or health regions, represent one approach to addressing these barriers. They tend to support managerial decision making and to focus on drugs or technologies. They are faster to execute than full HTAs and are contextualized for the setting from which they are produced [22]. These characteristics greatly improve uptake among decision makers, and mini-HTA initiatives have emerged in several tertiary care centers and urban regional health authorities in Canada [23]. However, producing mini-HTAs at the hospital/regional level requires resources and skills that may not be available in Canadian jurisdictions with fewer resources, e.g., rural and northern regional health authorities. Furthermore, mini-HTAs are limited in addressing the design and delivery of complex health services, e.g., community-based service models for seniors or the prevention and screening for type-2 diabetes, that are among the more pressing challenges of these same health jurisdictions.

The Contextualized Health Research Synthesis Program (CHRSP) of the Newfoundland and Labrador Centre for Applied Health Research (NLCAHR) has been specifically designed to incorporate these features to address the challenges noted above. CHRSP synthesizes high-level evidence (systematic reviews and health technology assessments), produces reports quickly, and, most importantly, optimizes the relevance of its products to the concerns and capacities of decision makers by building an ongoing partnership with high-level provincial health-system leaders, having them generate the research questions and, above all, tailoring the presentation of findings to a carefully developed understanding of the context(s) in which these decision makers must operate.

CHRSP was established in 2007 with the goal of increasing the use of health evidence by decision makers in the Newfoundland and Labrador health system. To build strong institutional support for the program, CHRSP partnered with the leaders of the province’s health system: the deputy ministers of key provincial government departments (namely, the Department of Health and Community Services and, beginning in 2016, the new Department of Children, Seniors and Social Development) and the Chief Executive Officers of the four provincial Regional Health Authorities.^1^ The CHRSP approach to the involvement of these knowledge users was not mere window dressing but an aggressive and comprehensive application of what the Canadian Institutes for Health Research have called “integrated Knowledge Translation” (iKT): “a way of doing research that involves decision makers/knowledge-users - usually as members of the research team - in all stages of the research process” [24, 25].

A key objective of CHRSP is to maximize the use of limited, locally available resources and expertise to synthesize existing systematic reviews on topics chosen by local knowledge users and to interpret the findings in light of local contextual factors.^2^ By working with decision makers, local researchers, and national experts, CHRSP has gradually developed a series of innovations to its methodology in an effort to become increasingly responsive to the needs of its health-system collaborators, more efficient in the production of its reports, and more effective at communicating results and promoting their uptake.

**Fig. 1:**
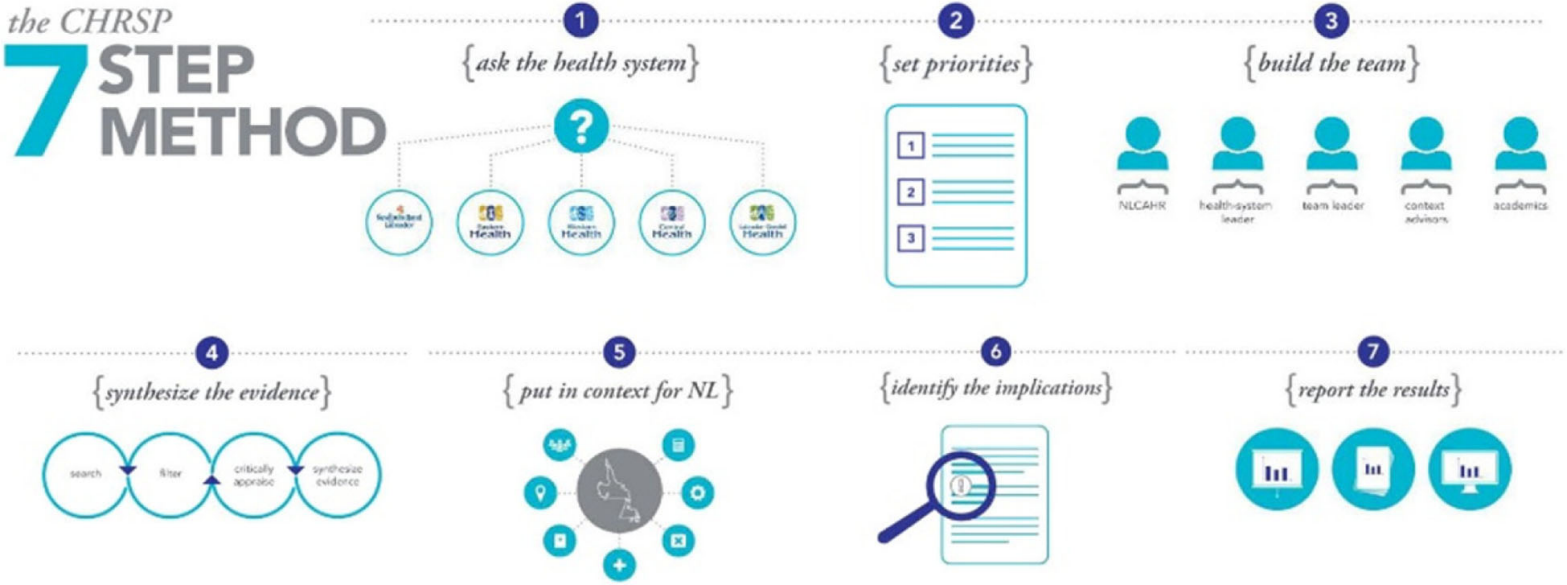
CHRSP 7-step method infographic

CHRSP now produces several types of reports. Our gold standard is the *Evidence in Context* (EiC) report which takes from 9 to 12 months to complete and follows seven steps, as outlined in Fig. 1 and detailed below [26].

## Methods

### Step 1: ask the health system

CHRSP consults directly with the leaders of the province’s six health-system organizations to identify upcoming decisions on practices, programs, or policies (“interventions”) that would benefit from evidence-based knowledge synthesis. From the beginning, the program has invested in relationship-building with these leaders to establish strong and ongoing collaboration. The commitment to, and active participation in, the program by these deputy ministers and CEOs are central to the program’s success.

#### CHRSP champions

In 2009, with funding from the Canadian Institutes for Health Research, CHRSP hosted a Knowledge Exchange Forum in which local health-system leaders met with leading Canadian experts in knowledge translation and evidence-based practice. The forum critically reviewed CHRSP’s processes and discussed lessons learned by similar research initiatives in other jurisdictions. The results were used to revise CHRSP methods, including its first major innovation—the introduction of “CHRSP champions,” whose role would be to serve as dedicated links between the healthcare system’s leaders and CHRSP researchers. Since that time, each of the six health-system leaders involved in CHRSP has been appointing a senior member or members of its staff to serve as the organization’s CHRSP champion(s). These champions are senior health-system managers with ready access to their system’s leader and a solid understanding of their healthcare organization and its catchment population. In each organization, they act on behalf of the leader to canvass the organization for issues for potential synthesis by CHRSP and to help the leader decide on which issues to submit for consideration.

### Step 2: set priorities

#### Compiling an annual list of research topics

Each year, CHRSP works with the leaders of the provincial healthcare system and their CHRSP champions to develop a slate of topics for study. While each organization has developed its own way of generating topics, in general the CHRSP champions will solicit potential topics from front-line workers, managers, administrators, decision makers, and health-system leaders (they may also provide their own topics for consideration) and pass the results to their organization’s leadership for review. On average, each organization submits 10–12 topics for consideration, thus generating a “long list” of potential studies. CHRSP filters this long list of submissions by:

- Consulting with CHRSP champions to clarify and refine the research questions;
- Consolidating duplicate and overlapping topics;
- Assessing submitted topics in terms of the feasibility of the topic for study using the CHRSP methodology and the appropriateness of the requested timelines; and
- Conducting preliminary literature searches to assess the scope and nature of available evidence.

Each organization’s submission to CHRSP includes an indication of its preference for timing, which will be considered when identifying which of our reporting formats is the most appropriate for each topic:

- An *Evidence in Context Report*—a comprehensive, in-depth examination of a topic, consisting of a 30–50-page report, a four-page summary, a one-page briefing note, and an online companion document for reference, and taking 9 to 12 months to complete; OR
- A *Rapid Evidence Report—*an expedited scoping review of the evidence on a topic, consisting of a 12–20-page report which requires 30 working days to complete.

At the time of writing, CHRSP is in the process of piloting a new product—*Jurisdictional Snapshot Reports—*which will provide decision makers with rapid jurisdictional scans of relevant policies, programs, or practices that have been implemented elsewhere and that might be considered for use in the province. This new product is described later in this article.

#### Supporting topic selection: guidelines and resources

CHRSP has developed a series of support tools and guidelines that explain, in plain language, the criteria for a feasible CHRSP topic as well as the processes involved in knowledge synthesis and contextualization. These tools help our system partners generate their long list submissions more effectively while enhancing their capacity to think of policy and program issues as potential research topics and developing a sympathetic understanding of the challenges faced by their research partners. The support tools include:

- Website materials and a champions’ handbook that highlight key aspects of CHRSP for knowledge users. These materials are publicly available to other members of the healthcare system, to local researchers, and to the public at large. They address how to identify and prioritize issues and how to set appropriate and realistic timelines. They outline how evidence is synthesized, how it should be interpreted, and how CHRSP builds its research teams [27].
- A standardized CHRSP topic submission form that guides respondents in conceptualizing, articulating, and operationalizing a potential CHRSP question. These include framing the issue as a research question using the PICOS framework,^3^ outlining the current state of the issue within the healthcare system context, identifying any approaches that have already been tried, and ruling out areas where study/evidence is not required.

These resources have been developed iteratively with our health-system partners and have provided an excellent opportunity for learning on both sides of the partnership. The use of these tools has helped our knowledge-user partners to increase their ability to think of issues as potentially researchable topics and to judge the usefulness and limits of research-based evidence for their decision-making processes. At the same time, developing and revising these resources has helped CHRSP researchers better appreciate the perspectives and needs of our health-system partners.

#### Consensus and priority-setting

The consolidated long list of topics is returned to the CHRSP champions and health-system leadership for them to review and rank. Each organization informs CHRSP of its top five topics. This vote yields a “short list” which forms the ballot for a second vote to determine a final list of seven topics ranked in order of priority for the next CHRSP research cycle. In addition to launching a new round of CHRSP projects, this priority-setting process gives health-system executives an opportunity to compare their challenges and priorities with those faced by their colleagues across the province. The process also provides the CHRSP research team with a privileged view of the current preoccupations of the province’s health-system leadership and an understanding of how these concerns develop over time.

### Step 3: build the team

A project team is assembled for each CHRSP project. What follows describes the composition of the teams for full *Evidence in Context* studies. For the shorter *Rapid Evidence Review* studies, a streamlined approach is used that will be described below.

For each *Evidence in Context* study, the team typically includes:

- An established national (or international) subject-matter expert who, through a short-term contract, agrees to provide guidance for setting the focus and scope of the project, interpreting the results of the synthesis, and structuring the report; the expert reviews all drafts of the report and participates in the main dissemination event;
- A health system leader (CEO, deputy minister, or senior delegate) who informs the project team about the background of the CHRSP topic and who helps to identify/delegate local health-system managers for the team;
- A health economist (when required) who provides support identifying, appraising, synthesizing, and interpreting evidence about economic dimensions of the topic, including likely costs, savings, and, benefits;
- Local co-investigators from the province’s university and healthcare system, including front-line workers, managers, decision makers, and members of community-based health organizations whose mandates have relevance to the topic. Local co-investigators are chosen from across the province in order to have appropriate geographic representation;
- A CHRSP staff project coordinator who is responsible for leading the project, including recruiting the expert, coordinating project team meetings and communications, developing search strategies, screening and filtering the results, data extraction and evidence synthesis, contextualization of the findings, and drafting the report;
- A health sciences librarian; and
- Other CHRSP staff and NLCAHR support personnel, including the CHRSP program director, who oversees all projects.

The project team works together throughout the CHRSP process—from the beginning to the end of each project. A CHRSP project officially starts with the first project team meeting at which time the focus and scope of the project, as well as any other parameters, are set by consensus. Team members are kept informed about research progress and preliminary results; they are consulted when challenges arise, and they are asked to review the report at three stages: evidence synthesis, contextualization, and final draft. The project team attends end-of-project dissemination events and members also assist within their organizations, wherever possible, in knowledge translation efforts to optimize the uptake and impact of the studies.

Each CHRSP project also involves context advisers and an external reviewer. Context advisors help the project coordinator to identify and explore possible contextual factors that could have an impact on the implementation of the findings. Local project team members may also serve as context consultants to provide input and data for the contextualization exercise. The external reviewer is a national/international subject matter expert who has not authored any of the evidence included in the synthesis. The external reviewer is also invited to participate in the end-of-project dissemination event.

For our *Rapid Evidence Reports*, the teams tend to be more compact, as will be explained below.

### Step 4: synthesize the evidence

Once a CHRSP project team has established the focus, scope, and eligibility criteria for studies (i.e., a suitable timeframe for publications that will be included and PICOS inclusion/exclusion conditions), CHRSP researchers search for systematic reviews (including meta-analyses and HTAs) in a limited number of the most relevant periodical indices and databases, including sources of gray literature.^4^ CHRSP staff also search for any recent primary research articles that would not yet have been captured by the existing systematic review literature.

CHRSP’s focus on systematic reviews is an approach that allows us to assess a large body of evidence relatively quickly and to deliver results more rapidly than most knowledge synthesis programs, without compromising scientific rigor. This makes the approach primarily meta-synthetic, producing systematic reviews of reviews or “overviews” as the Cochrane Collaboration calls them [28]. This scientifically robust approach allows us to deliver *Evidence in Context* reports in a timeframe that still qualifies them as “rapid reviews,” according to most definitions of the term [29–32].

In our *Evidence in Context* studies, the methodological quality of each systematic review being considered is assessed using the AMSTAR tool (Assessment of Multiple Systematic Reviews) [33, 34]. Each primary study is assessed using the Downs & Black checklist [35]. Critical appraisal is carried out independently by two CHRSP staff, and disagreements are resolved by discussion and consensus.

#### The CHRSP evidence rating system (ERS)

From the start, the CHRSP research team has experimented with different methods for rating the strength of the overall body of evidence for each particular intervention, working with decision makers and methodology experts to develop a system that is reliable and that has both internal and external validity. In 2015, based on criteria derived from the literature, and drawing on consultations, experience, and feedback from knowledge-user partners, CHRSP developed an Evidence Rating System to provide a robust and consistent approach.

The first step in the CHRSP ERS is to critically appraise eligible systematic reviews. AMSTAR scores are divided into three categories: high quality (70% or higher), moderate quality (between 40 and 69%), and low quality (less than 40%). Systematic reviews of low quality are excluded from the synthesis.

The second step is data extraction: the contents of retained systematic reviews are coded using the PICOS framework: Population, Intervention, Control, Outcome, and Setting. Only findings with matching PICOS parameters can be synthesized across multiple systematic reviews (i.e., CHRSP compares apples to apples and oranges to oranges). Individual PICOS-defined findings are also coded as being quantitative or qualitative, statistically significant or not, and as favoring the intervention being studied or the control group.

The third step in the CHRSP ERS is an enumeration of the primary literature that is covered by the systematic reviews for each PICOS-defined finding; when different systematic reviews synthesize the findings of the same primary research studies, this can result in the same evidence being “counted” more than once. When considering the strength of the body of evidence for a particular finding, this repeat counting may result in overestimating the strength of a body of evidence. In order to mitigate this possibility, the CHRSP ERS indexes the primary studies included within a given systematic review for each PICOS-defined finding. This index is then used to determine how many unique primary studies have been combined in the various systematic reviews addressing each PICOS-defined finding.

The CHRSP ERS combines the results of the above steps to establish a measure for the strength of the body of evidence for each PICOS-defined finding. This measure considers:

- The methodological quality of the systematic reviews;
- The number of unique primary research studies that underpin the findings; and
- The consistency of the review evidence (e.g., do all the high-quality systematic reviews report similar impact for a particular intervention on a particular outcome or do some disagree?)

The resulting evaluation of the body of evidence is reported using a five-point scale: Very Strong, Strong, Moderate, Weak, and Very Weak. The ERS is conservative by design, and our approach discounts any findings that are Weak or Very Weak.

The result is that a CHRSP meta-synthesis is highly specific (i.e., compares apples to apples), takes into account the methodological strengths and weaknesses of the systematic review evidence, assesses the true size of the evidence base (i.e., the number of individual studies involved), and emphasizes convergent research findings. Our knowledge users have indicated that this rating system makes intuitive sense to them and that its use in our reports provides them with a significant degree of confidence in the findings presented.

### Step 5: place the findings in context.

From the outset, each CHRSP project addresses two fundamental questions: “*What works?*” and “*What will work here?*” This approach acknowledges the “need for a two-pronged analysis, focused at once on the effects of the policy being studied and on the issues surrounding its implementation” [36].

The first question, “W*hat Works?”* is answered by the synthesis of research-based evidence. The second question, “*What will work here?”* requires an assessment of local contextual factors and their implications. A given contextual factor may have an impact on:

1. The *health equity* of an intervention, that is, the differential effectiveness either positive or negative, of an intervention for different groups in a population.
2. The *feasibility* of implementing an intervention, including costs, infrastructure, the recruitment and/or training of health human resources, and patient volumes.
3. The *acceptability* of an intervention from the perspective of relevant stakeholders, including decision makers, health service providers, political leaders, patients, caregivers, and families.

Health equity, feasibility, and acceptability are all critical considerations for decision makers [21]. Significant challenges in any of these areas can alter the suitability of a health practice, program, or policy [16]. Conversely, an intervention that is particularly beneficial for key groups within a population, with implementation requirements that are already in place (or that can be easily integrated into existing professional and patient/client patterns of behavior), might be seen as a better choice. Addressing the issue of context may be new to some researchers, but it is a significant concern to many others [7]. Contextual suitability has considerable impact on the willingness of decision makers to consider a recommended option [6] which is why we think it is critical for researchers to include a contextual lens in knowledge syntheses intended for decision makers [5, 37].

A distinctive feature of the CHRSP approach is that we address the issue of context explicitly and consistently in our reports. CHRSP interprets synthesis findings in light of local characteristics, capacities, and conditions that will have an impact on the implementation of health policy by the health decision makers of Newfoundland and Labrador and its four regional health authorities. We interview key informants from across the province including front-line healthcare workers, administrative data holders, community organizations, union representatives, managers, senior decision makers, patients, informal caregivers, and other appropriate stakeholders. Project team members are usually interviewed first and they suggest additional interviewees. Our consultations yield a series of potential contextual factors. For our *Evidence in Context* studies, if appropriate evidence is available for any particular contextual variable, CHRSP assesses its likely impact for our decision-making partners. If not, our reports frame the contextual factor as a question for consideration by decision makers. For our *Rapid Evidence Reports*, we compile a list of potential contextual factors that may influence how the intervention or interventions being considered are likely to work in our specific context but we do not analyze them in any detail.

CHRSP uses a framework that groups contextual factors thematically, as indicated in Table 1. Alternative taxonomies and additional factors are also conceivable.

**Table 1:** Contextual factor categories and examples

Contextual factor category	Examples of contextual factors to consider
Patient/client level	• Are there any epidemiological features or other features of the patient/client population that could affect equity of access, effectiveness, or appropriateness of the proposed intervention(s)? • Are there cultural elements that may enhance or detract from the expected effectiveness of the studied intervention?
Service/site design level	• How will any features of the site(s) of the proposed intervention affect its effectiveness or cost-effectiveness? • Is the design of the services feasible in the context of the existing infrastructure in some or all of the province’s regional health authorities?
Human resources	• Are there Health Human Resource (HHR) gaps? Does the province have the required number of appropriately trained and qualified practitioners to provide the service(s) in question? • Are there any alternative staffing arrangements or training options that could fill these HHR gaps?
Organization of health services	• Will the organization of existing front-line health services accommodate or conflict with the intervention/approach? • Can the existing management organization incorporate the intervention or will a significant reconfiguration be required?
Other departments/sectors	• Does the intervention require information, data or action from other government departments or provincial organizations, and will that information, data, or action be readily available? • Does the intervention require resources that are controlled by other government departments, other governments, or provincial non-governmental organizations?
Economic	• Are the existing financial incentives in the province consistent with the requirements of the studied intervention(s)? • How will the distribution of incomes affect the feasibility of delivering the studied intervention(s)?
Political	• What are the public/media expectations for the intervention? Are they realistic? • Is an intervention required as the result of a governmental decision or political pressure?

### Step 6: identify the implications for decision makers

The product of a CHRSP *Evidence in Context* project is a 30 to 50-page report. It includes brief sections on background, methods, and search results. The main body of the report focuses on the synthesis, the contextualization, and an analysis of the implications of these for the province’s decision makers.^5^ The project team identifies “key findings” from the evidence synthesis and highlights these at the beginning of each report. Key findings are the most *relevant* evidence synthesis findings and reflect the state of the available research. These key findings are then considered in terms of the contextualization results to come up with a list of the “implications for decision makers.”

For strategic reasons, CHRSP has opted to use the term “implications” rather than the more common “recommendations.” When we began our work, our Health System Partners were new to this type of decision support and we felt, correctly as it turned out, that they would be wary of “recommendations” that would appear to require action on their part. Using the term “implications” acknowledges that research-based evidence is only one of multiple types of input that health-system decision makers need to consider. CHRSP intended its reports to say: “here are things you should think about when considering this issue” rather than “this is the option you should choose when making a decision about this issue.”

Key messages and implications for decision makers are reviewed by the project team and by an external reviewer. The external reviewer is an acknowledged expert on the subject matter being examined who has been screened for potential conflicts of interest.^6^ He or she is contracted to review and critique the full report. Once we have a consensus, the full report and particularly the key messages and implications form the basis for a four-page executive summary and a one-page briefing note (see below).

### Step 7: report the results

#### Formats

*Evidence in Context* reports are published in three plain-language formats: no specialized expertise is required to understand the studies. The formats include a report of 30–50 pages (10–15 pages for *Rapid Evidence Reviews)*, a four-page summary, and a one-page briefing note for decision makers. Other online documents and presentations, press releases, and companion documents are also produced, as applicable.

#### Dissemination

The final report, the executive summary, and the one-page briefing note are delivered to the six Health System Partners for an embargo period of ten business days in order to give them time to prepare for its public release. All CHRSP project materials are then posted on the NLCAHR website and disseminated by direct e-mail to health system, community, and research groups across the province.

In addition, a dissemination event is organized to present the study to an invited audience. Each event is tailored to the individual project and ranges from a 2-h internally organized and supported meeting to day-long co-partnered and externally funded forums. The project’s subject-matter expert attends in person or via webinar and the external reviewer is invited (but not required) to attend. In addition to the project team, health-system administrators, managers, and front-line workers involved with the topic under study are invited to attend, as are academic researchers, students, and relevant patient/caregiver and community groups. Participants are encouraged to share invitations to open meetings to maximize impact beyond the immediate communication networks available to CHRSP.

In some cases, a separate dissemination event is organized that is intended exclusively for a particular sub-group; in other cases, we have organized a small group in camera session involving key decision makers, the subject expert, and CHRSP researchers. The key objective of these various dissemination events is to stimulate the use of CHRSP products and methods in decision making.

#### Public access to reports

*Evidence in Context* and *Rapid Evidence* reports are published on the NLCAHR website and placed in the Memorial University Libraries Research Repository, in Canada’s National Library and Archives and in Memorial University’s Yaffle Research Repository. They are also posted on the internal listservs of all Regional Health Authorities as well as in the Newfoundland and Labrador Medical Association’s Nexus Newsletter and the newsletter of the Association of Registered Nurses of Newfoundland and Labrador. Once a report has been disseminated, the NLCAHR team will solicit feedback from stakeholders and will host further meetings and events, as required, to help facilitate optimal uptake of the research results.

#### Feedback

Once sufficient time has elapsed between publication and the opportunity to apply the results (this depends on the topic and the complexity of the interventions under consideration), CHRSP solicits direct feedback from stakeholders and decision makers to evaluate uptake by asking how the reports have been used and to identify areas for potential improvement.

Now that the program has matured, the team is also beginning to update syntheses that are more than 5 years old in order to ensure that the original findings remain both current and relevant.

### Rapid evidence reports

In 2012, CHRSP introduced *Rapid Evidence Reports* to provide an expedited research-based decision-support product for decision makers. This report takes only 30 working days to produce after a consensus has been achieved among health-system partners and CHRSP researchers on the scope and wording of the research question. This expedited approach is chosen for topics for which our health-system partners have requested quick turnaround of the evidence to support a pending decision.

*Rapid Evidence Reports* provide a brief overview of the evidence in the systematic review literature and in primary studies not captured in the review literature. Each report includes a description of the scope of the research-based evidence, the strengths and gaps in the literature, and the principal areas of consensus, disagreement, and uncertainty in the research on the topic in question. *Rapid Evidence Reports* are carried out under the supervision and guidance of a key informant from the provincial health system and an external subject matter expert. *Rapid Evidence Reports* are not intended to provide an exhaustive synthesis of all the available literature, or to develop a systematic assessment of the methodological quality of the available research, or to thoroughly contextualize the findings of our literature scan. Rather, they provide decision makers with a reliable indication of the available research-based evidence and the core findings on the topic. If our key knowledge users find the contents of a given *Rapid Evidence Reports* suggestive but insufficiently comprehensive or authoritative, they can request that we carry out a full *Evidence in Context* study on the topic.

### Methodological developments

CHRSP also uses an integrated knowledge translation approach in the development of its methodology. From the beginning, the program has worked closely with knowledge users to identify opportunities for improvement. At the time of writing, CHRSP is developing two new innovations:

#### Introducing patient engagement

Starting in 2016, CHRSP has been consulting and planning to expand the scope of its integrated KT approach by developing a method for engaging patients and caregivers in the process. Adding patient/caregiver engagement will enhance CHRSP’s contribution to evidence-informed decision support by bringing people directly affected by healthcare policies and practices into the process, potentially broadening the scope of topics chosen and enhancing the range and the quality of the contextualization work involved. Patient and caregiver participants will work with CHRSP to make the design and dissemination of reports more accessible and relevant to lay audiences with diverse backgrounds and education levels.

#### New jurisdictional snapshot reports

In 2016–2017, CHRSP introduced *Jurisdictional Snapshot Reports* to provide decision makers with an overview of healthcare practices, programs, and policies from other jurisdictions. Jurisdictions are selected in consultation with decision makers and may include other provinces or regional health authorities in Canada, as well as other countries. These reports are intended to inform decision makers about the health policy landscape across jurisdictions with a focus not on research studies but on programs, tools, and other policy initiatives. Where possible, *Jurisdictional Snapshot Reports* will also clarify whether research-based evidence has been used to develop and/or evaluate the programs, tools, and policy initiatives involved. *Jurisdictional Snapshot Reports* may also help inform topic selection for subsequent CHRSP products, such as *Evidence in Context* or *Rapid Evidence Reports*.

## Results

As indicated in Table 2 (below), since 2007, CHRSP has completed 16 *Evidence in Context* reports, seven *Rapid Evidence Reports*, and one *Jurisdictional Snapshot Report*. These were based on a selection from approximately 125 topic submissions by the health system. At time of writing, our research team is at work on two *Evidence in Context* projects and one *Rapid Evidence Report*.

**Table 2:** Completed CHRSP projects, 2007–2017^7^

Evidence in Context (EiC) Reports (2007–2017)
1. Prevention and Screening for Type 2 Diabetes (2016) 2. Supporting the Independence of Persons with Dementia (2015) 3. Troponin Point-of-Care Testing (2014) 4. Agitation and Aggression in Residents with Dementia in LTC (2014) 5. Fall Prevention for Seniors in Institutional Healthcare Settings (2014) 6. Community-Based Service Models for Seniors (2013) 7. Telehealth for Specialist-Patient Consultations (2013) 8. Updated Evidence on Rural Dialysis Services (2013) 9. Age-Friendly Acute Care (2012) 10. Hyperbaric Oxygen Therapy for Difficult Wounds (2012) 11. Chronic Disease Management (2012) 12. Youth Residential Treatment (2010) 13. Reuse of Single-Use Medical Devices (2010) 14. Childhood Overweight and Obesity (2009) 15. PET-CT in Newfoundland and Labrador (2009) 16. Options for Dialysis Services in Rural and Remote Newfoundland and Labrador (2008)
Rapid Evidence Reports (RER) (2012–2017)
1. The Effectiveness of Digital Surveys for Collecting Patient Feedback (2016) 2. Reducing Wait Times for Outpatient Services (2016) 3. Health Promotion Strategies: Healthy Dietary Habits (2015) 4. Ambulatory Care Services for Patients with Chronic Heart Failure (2013) 5. Flu Vaccination for Healthcare Workers in Newfoundland and Labrador (2013) 6. Mobile Mental Health Crisis Intervention (2012) 7. Safe Patient Handling Programs and Injury Prevention (2012)
Jurisdictional Snapshot Reports (JS) (2017)
1. Identifying and Measuring Indicators that Place School-Aged Children/Youth at Risk of Poor Health Outcomes (2017)

### Evaluation

CHRSP recognizes that it takes time for its products to be taken up by the health system. Accordingly, we wait approximately 3 years before seeking feedback about whether and how our reports have been used. At that point, we solicit input from the following:

- Health System Leaders, CHRSP champions, project team members, and context advisors;
- All senior managers and administrators who were involved in the decision(s) related to the CHRSP project topic (including implementation);
- Community members who were involved in the project; and
- Everyone who attended a dissemination event.

CHRSP asks these participants two broad questions:

1. How was this report useful/relevant to your organization? Please tell us briefly how the report was considered or used in policy or practice decisions. Was it distributed within your unit, discussed at meetings, referenced in any briefs, incorporated into any decisions, added to your research library, etc.?
2. If the report was not useful or relevant, please tell us why not and how it might have been improved.

To date, CHRSP has received feedback on 12 of its published studies. Responses have been generally positive and constructive (see Table 3 below). Stakeholders of various types have indicated that they found CHRSP reports to be useful and relevant.

**Table 3:** Examples of CHRSP project feedback

Options for Dialysis Services in Rural and Remote Newfoundland and Labrador (2008)
“The research question was very appropriate and the results continue to help us to make decisions on dialysis. Combining the evidence with the contextualization made the results more useful to the system. Building the contextualization piece into the synthesis of the evidence made the report easier to read.” *Senior Official at Regional Health Authority*
Youth Residential Treatment (2010)
“The report has been an integral part of the planning and development phase for the Youth Treatment Centers that are being developed in this province – particularly in leading us to best practice material on programming and raising key questions and considerations. It continues to be referred to and referenced at both our provincial steering committee and local advisory committee levels, but most especially by me in my role within my health authority and the manager of the centre being developed by another provincial health authority, as we are the leads in terms of the nuts and bolts of planning and developing staffing, training, treatment modalities/programming and evaluation/outcome measures at the centers.” *Senior Official at Regional Health Authority*
Age-Friendly Acute Care (2012)
“St. Clare’s Hospital, a facility of Eastern Health, has just opened the province’s first Acute Care of the Elderly (ACE) unit. The evidence to support the effectiveness of ACE units was outlined in detail within the CHRSP study “Age-Friendly Acute Care” which was reviewed by health authorities across Newfoundland and Labrador in 2012.” *Research Analyst, Regional Health Authority*
Managing Agitation and Aggression in LTC Residents with Dementia (2014)
“This report gives me some good material to use when trying to convince nursing staff and administrators of the importance of the Music & Memory program we are implementing. I have shared this report with all Vice Presidents in my health authority and I like to quote your report by telling people that music ranks first on the list- so please know that your report has helped on a very real and tangible level. I will be using it when I try and expand the Music & Memory program to the provincial level next spring.” *Senior Administrator and Physician, Regional Health Authority*
Falls Prevention in Institutional Settings (2014)
“We used the results of this study to support a review of the existing falls prevention program and identification of opportunities to enhance the program at our health authority.” *Senior Official, Regional Health Authority*

CHRSP’s experience parallels and confirms the findings of much of the research literature on research-based decision support. Uptake of our reports, like that of similar knowledge synthesis products, is facilitated by direct and regular contact between researchers and health-policy decision makers [14]. Uptake is also facilitated by our contextualized approach that targets the needs, priorities, and capacities of those decision makers, emphasizing “pull” rather than “push” [11]. Our researchers have become more effective at informing health policy as we have deepened our understanding of the policy-making context [38] and as we have worked at making our products easier to read, with clear highlighting of the key findings [14, 18]. Above all, as the literature predicts, our fully integrated approach to KT, in which a regular group of knowledge users participates in the full range of our project activities, has proved to be an important factor in securing attention and uptake. [39–44].

## Discussion

CHRSP’s successes have come about by identifying and addressing challenges as they arise, but some challenges remain, not all of which can be readily remedied.

### Changes in personnel

As might be expected, there have been quite a few personnel changes in the staff of the Newfoundland and Labrador Centre for Applied Health Research and its CHRSP team. Since the NLCAHR is funded on annually renewable funding, all employees except the program director are employed on renewable 1-year contracts. As a result, there has been considerable staff rotation over the years. This has required the devotion of time and resources to maintaining the team’s institutional memory and its capacity to perform a specialized and technically demanding set of tasks.

Personnel changes at our health system partner organizations have been even more frequent, in terms of both health-system leadership and our CHRSP champions. These changes have sometimes resulted in delays in our topic selection process and have generated a need for repeated orientation exercises and training. Personnel changes at other levels of the provincial health system have created some challenges in our contextualization efforts, particularly when key contacts have left the organization and the system’s institutional memory has suffered.

### Organizational restructuring

Two principal reorganizations in Newfoundland and Labrador’s health system have occurred in the past decade. In 2004, the province reduced the number of regional health authorities from 14 to four, a transformation that made the initial development of CHRSP much easier. On the other hand, in 2014, the Department of Health and Community Services was split in two with the creation of a new Department of Seniors, Wellness and Social Development. Since the new department’s mandate aligned with many of the issues that CHRSP studies had covered in previous years, it was decided that its leader should be added to CHRSP and a new team of CHRSP Champions be recruited and trained. In 2016, the new department was renamed as the Department of Children, Seniors, and Social Development and its mandate changed once again, requiring further adaptation of the team and additional training. The possibility of even further restructuring of the province’s health system adds more uncertainty for CHRSP in terms of its partnership structure and its alignment with changing health-system priorities.

### Conflicting needs and interests

As the province’s health system, like its counterparts elsewhere in Canada and in other developed countries, comes under increasing fiscal and demographic pressures (tighter budgets, rapidly aging populations), our health-system partners have become increasingly eager for rapid turnaround in decision support. At the same time, their questions to CHRSP have become steadily more complex and multi-faceted. Further confounding the challenge of growing complexity, budgetary constraints have limited the ability of the NLCAHR to increase or even maintain its staff complement. As already noted, we have responded to time pressure by developing new, computer-aided processes for assessing the weight of evidence in our reports and have added two rapid response products, *Rapid Evidence Reports* and *Jurisdictional Snapshot Reports*, to our repertoire.

We have also sought, with varying success, to simplify complex questions by making a persistent effort to focus on the refinement of the research question during the initial stages of each study in an effort to keep the parameters of each study as well-delimited as possible. We have also eliminated one key CHRSP position, a full-time program manager, and transferred her responsibilities to the director of the NLCAHR who has taken on the role of program director for CHRSP.

### Balancing contexts

Given the multiple contexts of the province (indigenous and non-indigenous peoples, rural and urban healthcare settings, etc.), identifying “contextualization factors” can be a complicated task. The process of contextualization is further limited by the fact that our researcher teams cannot possibly locate and interview every appropriate contextual advisor from within such diverse communities. The fact that we must rely on a representative sample of informants means that placing the evidence in context may, or may not, reflect every contextual reality, even in such a small jurisdiction as Newfoundland and Labrador.

### Can CHRSP work elsewhere?

CHRSP has worked well in Newfoundland and Labrador for a variety of reasons:

- Newfoundland and Labrador differs in a variety of important ways from most other Canadian provinces and from the national and international jurisdictions where much of the available health research has been conducted. Decision makers in this province are keenly aware that the findings of much of this literature are, therefore, of limited or questionable applicability within the local context and are highly supportive of the local production of context-sensitive syntheses.
- The province’s health system is comparatively compact and involves a small number of key organizations whose leaders know one another and who are accustomed to working together. Securing their ongoing and active participation in CHRSP has thus proved easier than it might be in larger, more diverse jurisdictions.
- For a variety of reasons, this province’s health-system organizations have increasingly sought evidence to support their decisions and have striven to become learning organizations that are now skilled at generating “pull” for contextualized knowledge synthesis products.

Even in places where these factors do not pertain or are of lesser import, the CHRSP approach can still be useful. It appears particularly appropriate for jurisdictions (or parts of jurisdictions) in which contextual considerations are clearly necessary, for example in rural, remote, and northern regions in most Canadian provinces. The CHRSP methodology could also be used to contextualize a single set of findings for more than one context at a time, thus making it of greater potential interest to decision makers [16–20]. It is foreseeable that, provided a research question is of interest to decision makers in multiple jurisdictions, the findings of a synthesis done in one jurisdiction could be “re-contextualized” for decision makers in other jurisdictions. Teams working in other parts of Canada or in other countries could potentially use a similar approach to tailoring the findings of an evidence synthesis to the challenges and capacities of their own health systems.

The program is already expanding. Funding from the Manitoba Workers Compensation Board allowed the NLCAHR to collaborate with the research synthesis team at Toronto’s Institute for Work & Health on a project that examined the adaptation of the CHRSP methodology, in conjunction with the Institute’s own research synthesis program, for context-sensitive use in the field of occupational health and safety. Based on the report produced by that project, an Occupational Health and Safety model of the program is now being developed in Manitoba. In addition, working with a team of researchers and knowledge users in Northern Ontario and Northern British Columbia, CHRSP is now developing a proposal for a research program of integrated knowledge translation and contextualized evidence syntheses to support health decisions in various rural, northern, and remote regions of Canada.

## Abbreviations

AMSTAR: Assessing the Methodological Quality of Systematic Reviews
CEO: Chief executive officer
CHRSP: Contextualized Health Research Synthesis Program
EiC: Evidence in Context
ERS: Evidence rating system
HTA: Health technology assessment
KT: Knowledge translation
NLCAHR: Newfoundland and Labrador Centre for Applied Health Research
PICOS: Population, intervention, comparator, outcome, setting

## Footnotes

1 The province restructured their health care system in 2005 [45] to create four Regional Health Authorities: Eastern Health (which includes the tertiary care centers for the province in its capital, St. John’s), Central Health, Western Health, and Labrador-Grenfell Health, which includes all of sparsely populated Labrador.

2 Contextual factors for CHRSP include any variables involving feasibility, equity, cost, or acceptability that could have an impact on the local effectiveness and cost of a proposed policy, program, or practice.

3 Population, Intervention, Comparator, Outcome, and Setting parameters for the literature review [28].

4 Gray literature is non-commercially published materials, e.g., reports produced by government departments and agencies.

5 Each *Evidence in Context* project also produces an “Online Companion Document” that includes details of search strategies, search results, filtering and screening, critical appraisal, data extraction, analysis, and synthesis.

6 In particular, he or she should not have authored any of the systematic reviews included in the CHRSP study.

7
http://www.nlcahr.mun.ca/CHRSP/CompletedCHRSP.php.
